# Response Surface Optimization of Ultrasound‐Assisted Deep Eutectic Solvent Extraction of Bioactive Polysaccharides From Sword Bean (*Canavalia gladiata* (Jacq.) DC.) Pods

**DOI:** 10.1002/cbdv.71766

**Published:** 2026-10-21

**Authors:** Jieyu Xing, Linjing Zhang, Zhaoxuan Liang, Zheng Liu, Haiyun Chen, Xian Fu

**Affiliations:** ^1^ School of Pharmacy Guangdong Pharmaceutical University Guangzhou China; ^2^ School of Medicine Foshan University Foshan China; ^3^ Department of Neurology Shenzhen Bao'an District Songgang People's Hospital Shenzhen China

**Keywords:** antioxidant activity, anti‐neuroinflammation, *Canavalia gladiata* polysaccharides, hypoglycemic activity, sword bean

## Abstract

Safe and functional natural active ingredients are a research hotspot in early intervention studies of chronic diseases. This study focuses on a polysaccharide (CGPs‑1) from sword bean (*Canavalia gladiata* (Jacq.) DC.) pods. Its optimal ultrasound‐assisted extraction conditions were achieved with choline chloride/oxalic acid DES (2:1), 34% water, 21 mL/g, 65°C, 29 min at 280 W; the actual yield (33.24%) was consistent with the predicted value (33.96%). CGPs‑1 was identified as a pyran‑type polysaccharide, comprising rhamnose (Rha), glucuronic acid (GlcA), and galacturonic acid (GalA) in a molar ratio of 1.08:1:1.78. CGPs‑1 had IC_50_ values of 0.157 mg/mL (α‑glucosidase), 0.083 mg/mL (α‑amylase), 31.18 mg/mL (DPPH radicals), and 2.264 mg/mL (ABTS radicals), inhibited LPS‑triggered inflammatory response and LPS‑mediated neurotoxicity in BV2 cells, and also suppressed MPTP‐induced inflammatory response in zebrafish larvae. Results indicate that CGPs‑1 exhibits antioxidant, hypoglycemic, anti‑neuroinflammatory and neuroprotective potentials, providing preliminary experimental support for further research and the development of functional‑food‑related products derived from sword bean pods.

## Graphical abstract

The polysaccharide from sword been pods (CGPs‑1) demonstrates potent antioxidant activity through DPPH and ABTS radical scavenging (IC_50_: 31.18 and 2.264 mg/mL), inhibits key carbohydrate‑hydrolyzing enzymes including α‑amylase and α‑glucosidase (IC_50_: 0.083 and 0.157 mg/mL), reduces MPTP‐induced neuroinflammation in zebrafish embryos (0.1–0.4 mg/mL), suppresses LPS‑induced inflammatory responses in BV2 microglia, and protects neuronal cells against inflammatory toxicity, highlighting its promising potential for oxidative stress‐related and neuroinflammatory disorders.

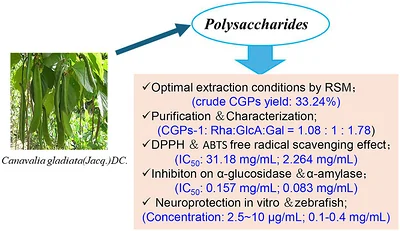

## 1 Introduction

Neurodegenerative diseases (e.g., Alzheimer's disease) are characterized by progressive neuronal loss, with complex pathogenic mechanisms and limited clinical intervention strategies, remaining an urgent health challenge awaiting breakthroughs. Driven by global aging and the impacts of modern lifestyles, the demand for brain health maintenance has become increasingly pressing, making natural active ingredients with both safety and functionality a research hotspot in early intervention studies. Accumulating evidence indicates that hyperglycemia, oxidative stress, and neuroinflammation are closely interrelated pathological factors, forming a vicious cycle that promotes the progression of neurodegenerative lesions [1, 2]. Peripheral metabolic disturbance can further exacerbate central nervous system injury via peripheral‑central crosstalk [2, 3, 4]. Chronic hyperglycemia induces excessive reactive oxygen species (ROS) production and sustained oxidative stress through mitochondrial dysfunction and polyol‐pathway activation. Excess ROS further activates NF‑κB and NOD‑like receptor thermal protein domain associated protein 3 (NLRP3) inflammasome signaling cascades, triggering microglial activation, and robust pro‐inflammatory cytokine secretion, which aggravates central neuroinflammation. In turn, persistent neuroinflammation weakens endogenous antioxidant defense capacity and exacerbates neuronal oxidative injury, ultimately elevating the risk of cognitive impairment and neurological dysfunction. As important components of natural products, medicinal plant polysaccharides have garnered extensive attention owing to their hypoglycemic, antioxidant and anti‑inflammatory properties, which are considered promising for early intervention against neurodegenerative risk factors [5, 6]; however, numerous untapped new plant‐derived polysaccharide resources remain.

*Canavalia gladiata* (Jacq.) DC., commonly referred to as sword bean, serves not only as a widely consumed vegetable but also as a recognized component of traditional Chinese folk medicine [7]. It has historically been employed for its purported therapeutic effects in alleviating inflammation, cancer, constipation, sinusitis, and pyogenic conditions [8]. Despite its extensive use, the precise mechanisms underlying these effects remain inadequately elucidated. Previous pharmacological studies explored multiple bioactivities of crude sword bean pod extracts via cell and animal models, including anti‑asthma [9], anti‐inflammatory [10], anti‑allergic [11], anti‑adipogenic [12, 13], and osteogenic [14] effects. Meanwhile, phytochemical investigations isolated and optimized extraction of non‑polysaccharide constituents such as tannins [15], lectins [16], polyphenols [17], terpenoids and flavonoids [18, 19]. Nevertheless, few of these existing studies have paid attention to sword bean polysaccharide fractions.

Polysaccharides are naturally occurring macromolecular polymers synthesized through the condensation and dehydration of more than ten monosaccharide units. Common techniques for plant polysaccharides include hot water extraction, enzymatic extraction, ultrasound‐assisted extraction, microwave‐assisted extraction, and supercritical CO_2_ extraction. Conventional hot water extraction suffers from prolonged extraction duration, high solvent consumption, low efficiency, and high operating temperature, which may cause structural degradation of thermally‑sensitive polysaccharides and impair their biological activity. Alternative approaches also exhibit inherent shortcomings: microwave‑assisted extraction may cause localized overheating and destroy polysaccharide backbones; enzymatic extraction is restricted by high reagent cost and narrow optimal reaction‑condition windows; supercritical CO_2_ extraction is hampered by costly instrumentation and poor solubility toward polar polysaccharides. Among these green extraction technologies, ultrasound‑assisted extraction relies on ultrasonic cavitation to disrupt plant cell walls under relatively mild conditions, accelerating the release of intracellular polysaccharides. Compared with high‐temperature extraction approaches, this technique requires shorter treatment time, reduces the risk of polysaccharide thermal decomposition, and better preserves the bioactivity of target polysaccharides [20, 21]. This mild extraction strategy has also been proven effective for harvesting bioactive plant polysaccharides in recent ultrasonic extraction research [22]. Accordingly, ultrasonic‑assisted extraction was selected to isolate polysaccharides from s‑word bean pods in the present study. Nevertheless, water‑based extraction solvents still suffer from drawbacks such as long extraction time, loss of active components, low extraction efficiency and large solvent consumption [23, 24]. Therefore, exploring novel green solvents to improve extraction yield and efficiency has attracted considerable research attention. In line with the concept of safety and environmental friendliness, deep eutectic solvents (DESs) have emerged as promising alternatives. DESs are constructed via hydrogen bond interactions between hydrogen bond acceptors and hydrogen bond donors. Featuring high extraction efficiency, environmental benignity, and strong solubilizing capacity for diverse bioactive substances, they have been widely applied to the extraction of plant derived active constituents [25]. Generally formulated by mixing two‑ or three‑component ingredients, DESs exhibit melting points markedly lower than that of any individual constituent. Choline chloride (ChCl) and betaine are frequently used hydrogen‑bond acceptors, while amides, alcohols, carboxylic acids and saccharides can serve as hydrogen‑bond donors, such as urea, ethylene glycol, glycerol, citric acid, lactic acid, and glucose. For preparation, hydrogen‑bond acceptors and donors with a specific molar ratio are heated and stirred until a homogeneous transparent liquid is obtained. A small amount of water is often incorporated into the system to reduce viscosity, improve mass transfer of target substances, and facilitate subsequent separation [26, 27].

The present work intended to establish an efficient ultrasound‑assisted deep eutectic solvent extraction procedure for *C. gladiata* polysaccharides (CGPs), investigate their basic structural characteristics, and preliminarily evaluate their hypoglycemic, antioxidant, and anti‑neuroinflammatory bioactivities, which could offer experimental reference for future application of sword‑bean‑derived polysaccharides. To achieve this goal, single‑factor and response‑surface methodology was adopted for extraction condition optimization. Crude CGPs were further purified by DEAE‑Sepharose and Sephadex G‑75 chromatography. Structural characterization included molecular‑weight determination, monosaccharide composition analysis, and Fourier transform infrared (FTIR) spectroscopy. Multiple in vitro assays together with a zebrafish model were employed to assess the biological activities of purified CGPs.

## 2 Results and Discussion

### 2.1 DESs Screening

Ultrasound‑assisted DES extraction was applied to obtain crude polysaccharides from sword bean pods. A standard curve of glucose concentration (C)—absorbance (A) was drawn and used to calculate the concentration of polysaccharides in the polysaccharide yield (*A* = 3.6643C–0.0111, *R* ^2^ = 0.9991). Polysaccharides are biological macromolecules formed by multiple monosaccharide molecules connected through glycosidic bonds, and their solubility varies greatly. A set of preliminary experimental parameters was fixed during DES screening to exclude the interference of other variables and focus on solvent‐dependent extraction performance. Thus, under conditions of water content of 30%, liquid–solid ratio of 20 mL/g, ultrasonic power of 280 W, ultrasonic time of 30 min, and temperature of 65°C, seven DESs (all used ChCl as Hydrogen Bond Acceptor, HBA) were selected for the screening. As shown in Figure 1a, the polysaccharide yield showed a clear pattern: the yields in descending order were carboxylic acid–based DES–1 (oxalic acid, OA), DES–7 (malonic acid, MA), DES–3 (citrate, CA); amide–based DES–6 (urea); and polyol–based DES–4 (glycerol, Gly), DES–2 (1,4–butanediol, BDO), DES–5 (ethylene glycol, EG). Different DES compositions lead to distinct properties, such as polarity, solubilizing capability, viscosity, and fluidity [28]. This observation is consistent with previous reports that carboxylic‑acid‑based choline‑chloride DESs generally exhibit superior extraction performance for plant‑derived polysaccharides [22, 29]. The underlying mechanisms can be partly explained by the physicochemical features of carboxylic–acid–based DESs described in earlier investigations [30]. Compared with polyols‑based DESs and amide‑based DESs, carboxylic–acid–based DES has strong polarity and ionic‐bonding effects. The carboxyl group can dissociate into a negative charge, forming ion pairs with cations in DES (such as choline cations), providing a large number of hydrogen–bond donors (hydroxy + carboxyl). Its choline cation can bind to the carboxyl group of acidic polysaccharides through ionic bonds, significantly enhancing solvent affinity towards polysaccharides. It can also more effectively destroy the intra– /inter– molecular hydrogen bonds of neutral polysaccharides and promote their dissolution. The acidity of carboxylic acids facilitates cell–wall dissociation and may denature proteins, reducing their encapsulation or binding to polysaccharides. At the same extraction temperature (such as 50°C–60°C), carboxylic–acid–based DES with low melting point exhibit lower viscosity, better fluidity, and a faster diffusion rate of solvent molecules, which is conducive to polysaccharide dissolution. By comparison, polyol‑ and amide‑based DESs displayed higher viscosity under the tested conditions, which hampered mass‑transfer. Moreover, they provided weaker ionic‑interactions with acidic polysaccharide fractions, thus yielding relatively lower polysaccharide contents.

**FIGURE 1:**
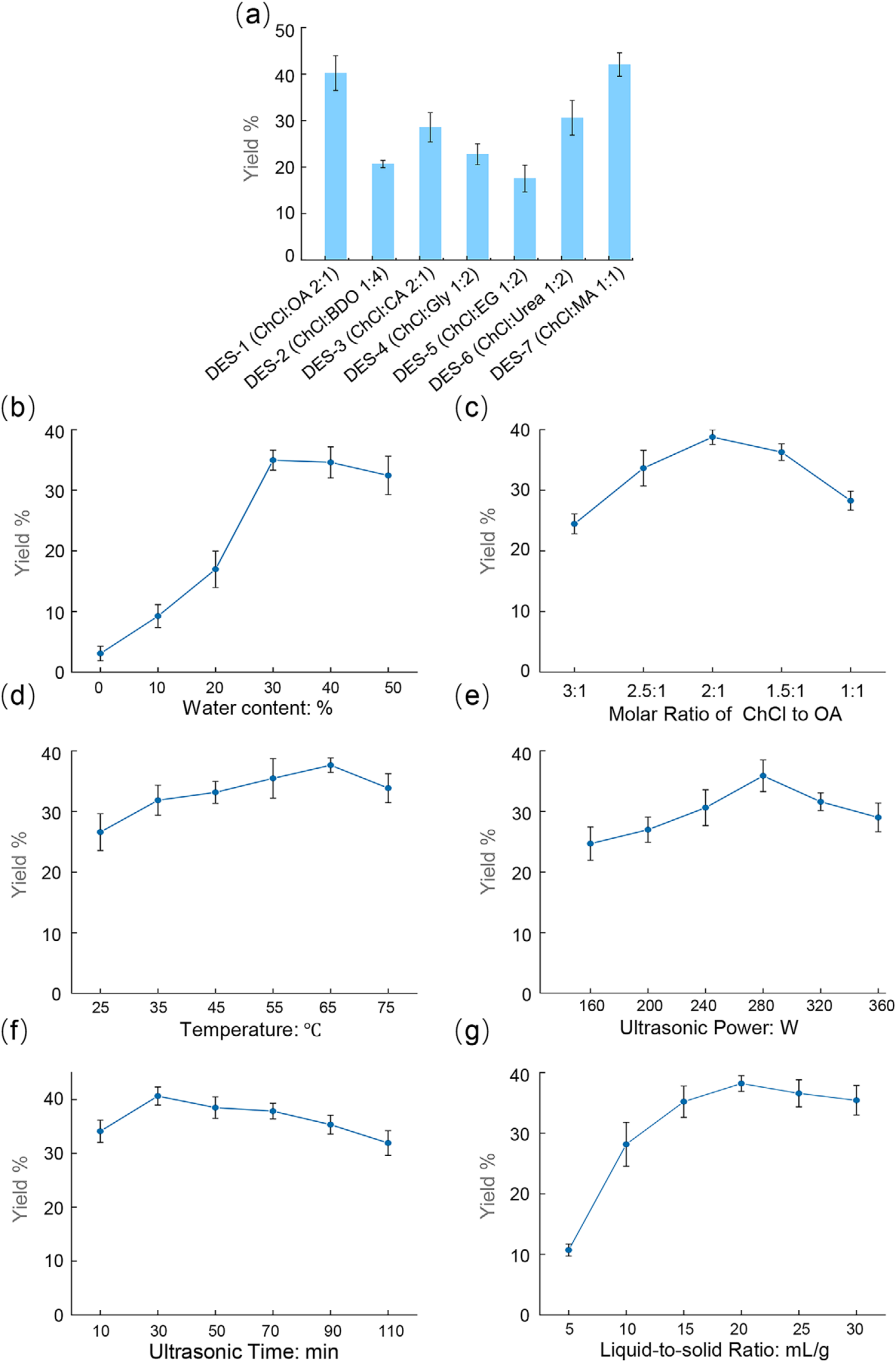
Results of single factor experiments. The effect of different DESs (a), water content (b), different molar ratios of DES (c), temperature (d), ultrasonic power (e), ultrasonic time (f), and liquid–to–solid ratio (g) on the polysaccharide yield of crude CGPs.

The optimal polysaccharide yield was achieved using the DES–1 of ChCl–OA (2:1) system. Therefore, DES–1 was selected as the solvent for subsequent experiments.

### 2.2 Single Factor Experiments

Next, with the screened DES‑1 (ChCl‑OA) as extraction medium, the effects of various parameters, including DES water content and DES molar ratio, extraction temperature, ultrasonic power, ultrasonic time, and liquid‑to‑solid ratio, on polysaccharide yield were systematically evaluated through sequential single‑factor experiments. The optimal level obtained from each single‑factor assay was adopted as the fixed condition for the subsequent parameter investigation, and this procedure was repeated for all tested variables.

When investigating water content, other parameters were fixed as DES‑1 (ChCl‑OA in the ratio of 2:1), liquid‑to‑solid ratio of 20 mL/g, ultrasonic power of 280 W, ultrasonic time of 30 min, and temperature of 65°C. As shown in Figure 1b–g, polysaccharide yield initially increased and then decreased with the continuous change of each variable, showing a distinct peak value for every factor. The corresponding optimal conditions were as follows: DES water content peaked at 30%, DES molar ratio (ChCl‐OA) peaked at 2:1, extraction temperature peaked at 65°C, the ultrasonic power peaked at 280 W, ultrasonic time peaked at 30 min, and the liquid‑to‑solid ratio was at 20:1 mL/g. Accordingly, these optimal single‑factor conditions were selected for the following optimization experiments.

The observed trend of initial increase followed by decrease in polysaccharide yield under varying conditions is commonly reported in ultrasound‑assisted DES extraction. Appropriately elevated temperature, ultrasonic power and prolonged sonication time facilitate cavitation and mass transfer, thus promoting polysaccharide dissolution. Nevertheless, excessive temperature or ultrasonic energy may cause partial degradation of extracted polysaccharides, leading to reduced yield [22]. Excess water diluted the DES system and weakened its solubilizing capacity toward polysaccharides, whereas insufficient water resulted in high DES viscosity and poor mass‑transfer efficiency.

It should be noted that sequential single‐factor strategy adopted in this work optimizes one variable at a time while fixing others at the previously obtained optimal levels. This approach may yield path‑dependent results when significant interactions exist among variables, meaning that the derived optimal levels could be affected by the order of factor investigation. Therefore, response‑surface methodology was further applied for multi‑variable optimization.

### 2.3 Response Surface Experiments

Based on the results of single‑factor experiments, polysaccharide yield (Y) was set as the response value. Four key variables, namely DES water content (A), liquid‑to‑solid ratio (B), ultrasonic time (C), and ultrasonic power (D) were selected for response‑surface optimization. Three levels were assigned to each factor, with the single‑factor‑derived optimal condition set as the central point. The Box–Behnken design (BBD) was employed with Design‑Expert 13.0 software to evaluate variable interactions and determine the global optimal extraction conditions. Table 1 lists the coded and actual levels of independent variables as well as the corresponding response values.

**TABLE 1:** Box‑Behnken design matrix and corresponding experimental results.

Run	Water content (%)	Liquid‑to‑solid ratio (mL/g)	Ultrasonic time (min)	Ultrasonic power (W)	Yield (%)
1	40	20	50	280	27.58
2	40	20	30	240	13.89
3	30	20	30	280	33.71
4	20	20	50	280	24.08
5	30	15	10	280	28.42
6	20	25	30	280	21.95
7	30	20	10	240	13.48
8	20	15	30	280	25.2
9	30	20	10	320	15.81
10	20	20	30	320	11.44
11	40	20	30	320	16.65
12	30	25	50	280	30.12
13	40	20	10	280	31.51
14	40	25	30	280	32.25
15	20	20	10	280	18.27
16	30	20	50	240	14.94
17	30	15	50	280	27.72
18	30	20	30	280	34.15
19	30	15	30	240	16.34
20	30	25	10	280	29.17
21	30	20	30	280	33.15
22	30	20	30	280	32.15
23	30	25	30	240	20.13
24	40	15	30	280	30.89
25	30	15	30	320	18.68
26	30	20	50	320	16.97
27	30	25	30	320	14.62
28	30	20	30	280	33.42
29	20	20	30	240	12.26

Table 2 presents the analysis results. According to the regression analysis, a quadratic polynomial model was established to describe polysaccharide yield. *R* ^2^ = 0.9829, indicating a good model fit. The coefficient of variation (C.V.%) was 6.16, demonstrating acceptable experimental precision. The model *p*‑value revealed that the regression equation for polysaccharide yield was significant (*p* < 0.05), while the lack‑of‑fit term was non‑significant (*p* > 0.05), suggesting adequate fitting performance for prediction of polysaccharide yield. Based on *p*‑values, the terms A, AC, BD, A^2^, C^2,^ and D^2^ exerted significant effects on polysaccharide yield (*p* < −0.05), whereas other coefficients were insignificant. Based on *F*‑values, the factor influence order on sword bean polysaccharide yield was: water content (A) > ultrasonic time (C) > ultrasonic power (D) > liquid‑to‑solid ratio (B).

**TABLE 2:** Analysis of variance for the quadratic regression model.

Source	Sum of Squares	DF	Mean Square	*F*‐value	*p*‐value	Significance
Model	1668.85	14	119.20	57.32	< 0.0001	Significant
A (Water content)	130.48	1	130.48	62.74	< 0.0001	
B (Liquid‐to‐solid ratio)	0.0817	1	0.0817	0.0393	0.8458	
C (ultrasonic time)	1.88	1	1.88	0.9041	0.3578	
D (Ultrasonic power)	0.8164	1	0.8164	0.3926	0.5410	
AB	5.31	1	5.31	2.55	0.1323	
AC	23.72	1	23.72	11.40	0.0045	
AD	3.20	1	3.20	1.54	0.2349	
BC	0.6806	1	0.6806	0.3273	0.5763	
BD	15.41	1	15.41	7.41	0.0165	
CD	0.0225	1	0.0225	0.0108	0.9186	
A^2^	147.00	1	147.00	70.69	< 0.0001	
B^2^	7.43	1	7.43	3.57	0.0796	
C^2^	68.43	1	68.43	32.90	< 0.0001	
D^2^	1431.48	1	1431.48	688.34	< 0.0001	
Residual	29.11	14	2.08			
Lack of Fit	26.87	10	2.69	4.78	0.0726	Not significant
Pure Error	2.25	4	0.5622			
Cor Total	1697.96	28				
R^2^	0.9829					
R^2^_adj_	0.9657					
C.V.%	6.16					

Three‑dimensional (3D) response‑surface and contour plots were generated to visualize variable‑to‑variable interactions for polysaccharide yield (Figure 2a–f). Generally, steeper response‑surface curvature and elliptical‑shaped contour lines indicate stronger interaction between two factors, whereas near‑circular contours suggest weak interaction. As observed in Figure 2, polysaccharide yield first increased with rising variable levels and then decreased upon further elevation of each factor, which agreed well with the trends obtained from single‑factor experiments. The fluctuation magnitude of 3D response‑surface curvature matched the foregoing ANOVA statistical outputs. Elliptical contour shapes confirmed significant two‑way interactive effects between certain pairs of variables on polysaccharide yield, which was supported by their corresponding *p*‑values from ANOVA. By contrast, approximately circular contours implied negligible interactions for other factor pairs.

**FIGURE 2:**
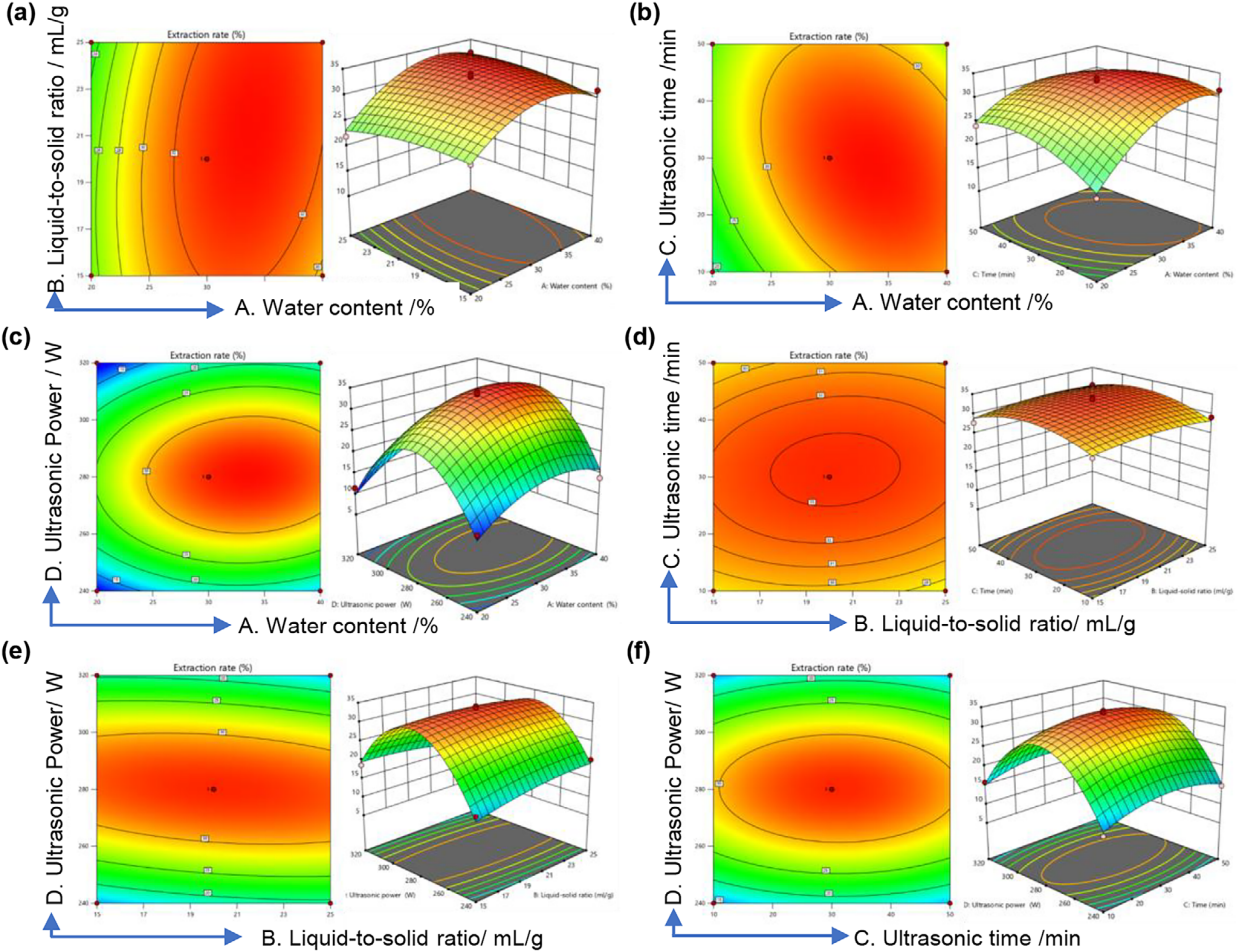
3D response surface plots and contour plots showed the inter‐effects on the polysaccharide yield from sword bean pods. (a) water content of DES and liquid‐to‐solid ratio water, (b) water content of DES and ultrasonic time, (c) water content of DES and ultrasonic power, (d) liquid‐to‐solid ratio and ultrasonic time, (e) liquid‐to‐solid ratio and ultrasonic power, and (f) ultrasonic time and ultrasonic power.

Specifically, the interaction between DES water content (A) and ultrasonic time (C) exerted the most prominent synergistic effect, which arose from the combined effects of decreased DES viscosity and ultrasonic cavitation that collectively facilitate polysaccharide dissolution from plant matrix [22, 31]. Meanwhile, the interaction between liquid‑solid ratio (B) and ultrasonic power (D) was also significant. A reasonable explanation is that sufficient solvent supply together with appropriate ultrasonic energy jointly promotes mass transfer of polysaccharides [22, 27]. By comparison, several remaining factor‑pair interactions were non‑significant within the tested ranges.

The final quadratic polynomial regression equation for predicting polysaccharide yield from sword bean pods is as follows:

(1) $$\begin{array}{ccc} Y & = & 33.32+3.30A+0.0825B+0.3958C+0.2608D\\ & & +1.15\mathrm{AB}-2.44\mathrm{AC}+0.8950\mathrm{AD}+0.4125\mathrm{BC}-1.96\mathrm{BD}\\ & & -0.075\mathrm{CD}-4.76A^{2}-1.07B^{2}-3.25C^{2}-14.86D^{2} \end{array}$$

By analyzing the regression equation, the optimal extraction conditions were as follows: liquid‑to‑solid ratio of 21.1527 mL/g, DES water content of 33.9375%, ultrasonic time of 28.5648 min, and ultrasonic power of 280.233 W, yielding a predicted polysaccharide yield of 33.9604%. For practical application, these conditions were adjusted to a liquid‑to‑solid ratio of 21 mL/g, DES water content of 34%, ultrasonic time of 29 min, and ultrasonic power of 280 W, and the actual polysaccharide yield was 33.2435%, showing good agreement between predicted and experimental values and confirming the reliable fitness of the regression model. Meanwhile, ultrasound‑assisted water extraction was carried out under the same conditions for comparison, giving a polysaccharide yield of only 28.67%. The result demonstrated the superiority of the DES‑based extraction strategy under the present experimental conditions. The optimized DES extraction parameters could provide a reference for further scale‑up investigation of polysaccharide preparation from sword bean pods.

### 2.4 Analysis of Gradual Purification

In addition, anion‑exchange (DEAE‑Sepharose) chromatography combined with gel filtration (Sephadex G‑75) chromatography was applied for the sequential purification of crude polysaccharides. Crude CGPs first underwent anion‑exchange separation using a DEAE‑Sepharose column, yielding acidic polysaccharides within the 0∼0.8 M NaCl gradient, as shown in Figure 3a. Further separation using a Sephadex G‐75 column yielded a single symmetrical peak, designated as CGPs‑1, as shown in Figure 3b. The polysaccharide purity, defined as total sugar content in the dry sample, reached 93.75% for CGPs‑1, compared with 78.94 % for the crude sample before purification. Consistent with the previous report [32], such two‑step chromatographic purification effectively elevated polysaccharide purity. The elution behavior on DEAE‑Sepharose indicated that the target fraction belonged to an acidic polysaccharide. A single symmetric peak on Sephadex G‑75 suggested relatively good molecular‑weight homogeneity of the obtained CGPs‑1, which was suitable for subsequent structural characterization and bioactivity evaluation.

**FIGURE 3:**
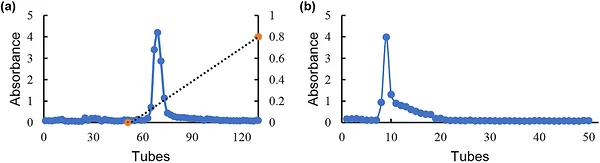
Elution profiles of polysaccharides from sword bean pods during two‑step chromatographic purification. (a) Crude CGPs on a DEAE‑Sepharose column; (b) CGPs‑1 on a Sephadex G‑75 column.

### 2.5 Molecular Weight Determination

As shown in Figure 4a, the HPGPC profile of CGPs**‑**1 displayed a single main elution peak. The weight**‑**average molecular weight (Mw) of CGPs‑1 was determined to be 274.063 kDa. The single‑peak feature indicated that CGPs‑1 possessed relatively good molecular‑weight homogeneity. To further illuminate its structural features, the monosaccharide composition was subsequently analyzed by HPLC.

**FIGURE 4:**
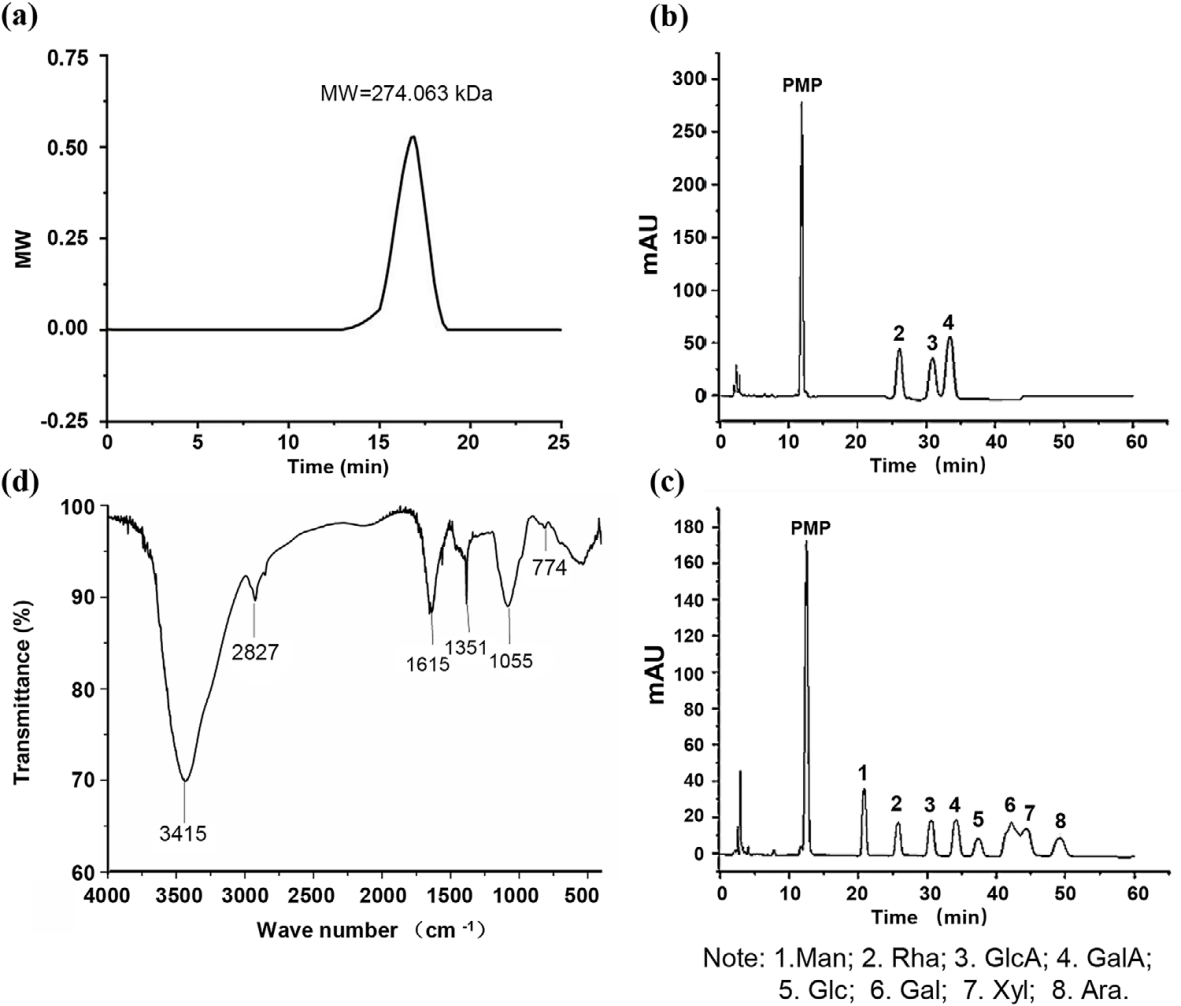
Structural characterization of CGPs‑1: (a) HPGPC molecular‑weight profile, (b) HPLC profile of monosaccharide composition, (c) HPLC profile of mixed standard monosaccharides, and (d) FTIR spectrum.

### 2.6 Analysis of Monosaccharide Components

As shown in Figure 4b,c, CGPs‑1 was composed of rhamnose (Rha), glucuronic acid (GlcA), and galacturonic acid (GalA). After correction using standard monosaccharides, the corresponding molar ratio was calculated as 1.08:1:1.78. Galacturonic acid occupied the highest molar proportion among the detected monosaccharide components, indicating that galacturonic acid is the predominant monosaccharide unit within CGPs‑1. The compositional analysis also revealed abundant uronic‑acid moieties (glucuronic acid and galacturonic acid) in CGPs‑1.

### 2.7 Analysis of FTIR

As shown in Figure 4d, CGPs‑1 exhibited characteristic absorption peaks at 3415 cm^−^^1^ (O─H stretching vibration), 2827 cm^−^^1^ (C─H stretching vibration), 1615 cm^−^^1^ (asymmetric stretching vibration of carbonyl groups), 1351 cm^−^^1^ (C─H angular deformation vibration), 1055 cm^−^^1^ (C─O stretching vibration, characteristic peak of pyranose sugars), and 774 cm^−^^1^ (out‑of‑plane bending vibration of C─H). These spectral features confirmed typical structural signatures of polysaccharides. The absorption band near 1055 cm^−^^1^ suggested the existence of pyranose‑type ring structures within CGPs‑1. Moreover, the signal at 1615 cm^−^^1^ further supported that CGPs‑1 was an acidic polysaccharide, which was consistent with the elution behavior observed in the anion‑exchange chromatography as well as the foregoing monosaccharide compositional data. Collectively, the FTIR spectra offered preliminary structural information for CGPs‑1. Further comprehensive structural characterization, such as methylation and NMR analysis, is required to elucidate its precise backbone configuration.

### 2.8 In‑Vitro Hypoglycemic‑Related Activity‐

The in‑vitro hypoglycemic‑related potential of CGPs‑1 was evaluated by determining the inhibitory effects against α‑glucosidase and α‑amylase. As shown in Figure 5a, the α‑glucosidase inhibitory effect of CGPs‑1 increased in a concentration‑dependent manner, with an IC_50_ of 0.157 mg/mL against α‑glucosidase. Similarly, CGPs‑1 exhibited concentration‑dependent α‑amylase inhibition (Figure 5b), yielding an IC_50_ of 0.083 mg/mL. The results showed that CGPs‑1 demonstrated α‑glucosidase and α‑amylase inhibition but at a much weaker level than acarbose in terms of mass concentration. CGPs‑1 was more effective in inhibiting α‑amylase than α‑glucosidase. For comparison, CGPs‑1 exhibited moderate α‑glucosidase inhibitory potency, with its activity inferior to polysaccharides PR‑1 and PR‑2 from *Rosa roxburghii* Tratt. (reported IC_50_: 0.049 mg/mL and 0.057 mg/mL, respectively) [33]. By contrast, CGPs‑1 displayed much stronger inhibitory capacity than polysaccharides (GFP) derived from ginseng folium (IC_50_> 3 mg/mL) [22]. Collectively, CGPs‑1 exhibited favorable in‑vitro inhibitory potential against carbohydrate‑digesting enzymes.

**FIGURE 5:**
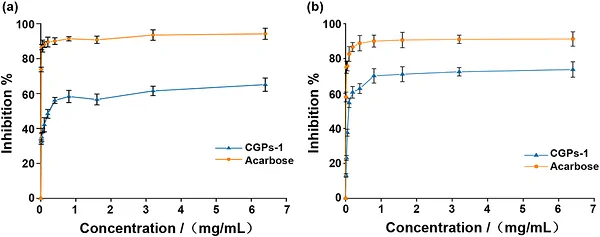
Inhibitory activities of CGPs‑1 against carbohydrate‑hydrolyzing enzymes. (a) α‑glucosidase inhibitory activity; (b) α‑amylase inhibitory activity.

### 2.9 In‑Vitro Antioxidant Activity

As shown in Figure 6a, CGPs‑1 exhibited concentration‑dependent DPPH• radical‑scavenging capacity, with an IC_50_ of 31.18 mg/mL. As shown in Figure 6b, CGPs‑1 also showed concentration‑dependent ABTS•^+^ radical‑scavenging effect, yielding an IC_50_ of 2.264 mg/mL. Although CGPs‑1 could scavenge both DPPH• and ABTS•^+^ scavenging effects, its performance was considerably weaker than the positive control VitC at equivalent mass concentration. For comparison, CGPs‐1 was markedly less potent than *Tricholoma lobayense* polysaccharides (TLP), whose reported scavenging rates ranged from 3.44% to 93.47% over the concentration range of 2–10 mg/mL [34]. Besides, according to IC_50_ data, CGPs‑1 was more efficient toward ABTS•^+^ than DPPH• radicals, and highly consistent with polysaccharide from *Brasenia schreberi* (BPL‑2), which displayed an ABTS•^+^ radicals scavenging IC_50_ of 0.239 mg/mL and a DPPH• radical scavenging IC_50_ of 1.863 mg/mL [35], indicating that uronic acid moieties are the core contributor to ABTS antioxidant performance, while DPPH activity is jointly regulated by solvent compatibility and molecular conformation [22]. Collectively, CGPs‑1 possessed certain in‑vitro free‑radical‑scavenging potential.

**FIGURE 6:**
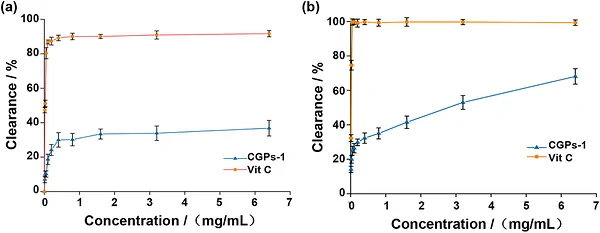
In‑vitro antioxidant activity of CGPs‑1. (a) DPPH**•** scavenging activity; (b) ABTS**•** ^+^ scavenging activity.

### 2.10 Inhibitory Activity on Inflammation in LPS‑Induced BV2 Cells

To explore the impacts of CGPs‑1 on LPS‑stimulated pro‑inflammatory reactions, BV2 microglial cells were pre‑incubated with CGPs‑1 (2, 5, 10 µg/mL) for 2 h before stimulation with 1 µg/mL LPS for 24 h, and CCK‑8 viability assays were subsequently performed. As shown in Figure 7a, neither 1 µg/mL LPS alone nor its combination with CGPs‑1 (2, 5, 10 µg/mL) produced obvious cytotoxicity (*p* > 0.05). Therefore, the levels of nitric oxide (NO) and pro‑inflammatory cytokines released into cell culture supernatants were further determined. As shown in Figure 7b, LPS notably promoted NO secretion in BV2 cells (*p* < 0.001). In contrast, CGPs‑1 remarkably suppressed LPS‑triggered NO generation in a concentration‑dependent manner. Simultaneously, as shown in Figure 7c–e, LPS significantly elevated the release of pro‑inflammatory cytokines (TNF‑α, IL‑1β and IL‑6) in BV2 cells (*p* < 0.001), whereas CGPs‑1 inhibited the secretion of these cytokines also in a concentration‑dependent manner. Collectively, these results indicate that CGPs‑1 exerted an inhibitory effect on the pro‑inflammatory response in LPS‑stimulated BV2 microglial cells.

**FIGURE 7:**
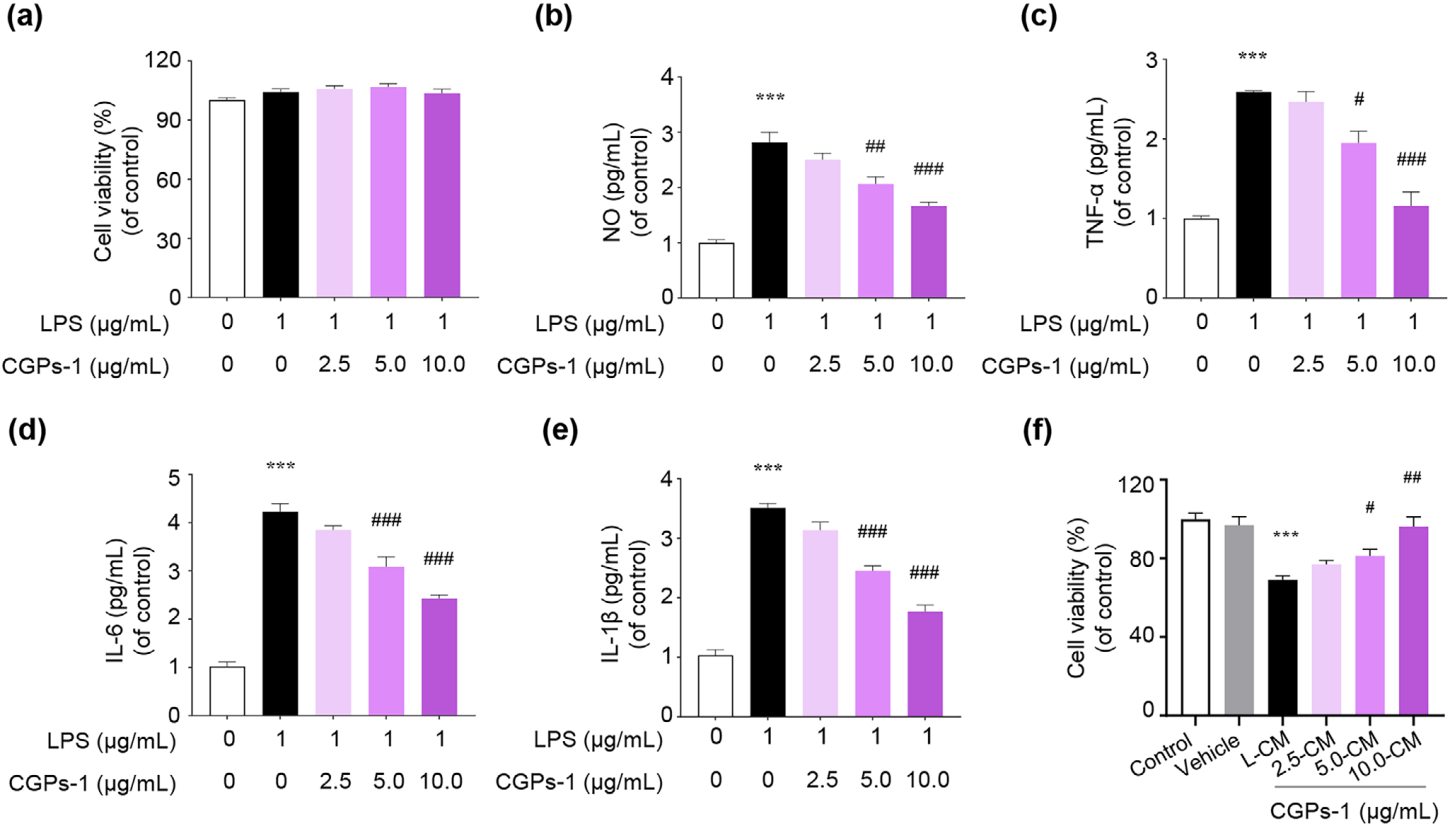
Inhibitory activities of CGPs‐1 on inflammatory response in LPS‐induced BV2 microglial cells. (a) Cell viability; (b) NO release; (c) TNF‑α secretion; (d) IL‑6 secretion; (e) IL‑1β secretion; (f) Viability of N2a cells exposed to BV2‑derived conditioned medium. *p < 0.05, **p < 0.01, ***p < 0.001 vs. control group; ^#^ *p* < 0.05, ^##^ *p* < 0.01, ^###^ *p* < 0.001 vs. LPS group.

To further assess whether CGPs‑1 could alleviate microglia‑mediated neuronal injury, N2a cell and conditioned medium (CM) from LPS‑activated BV2 cells were adopted. As shown in Figure 7f, LPS‑treated conditioned medium (L‑CM) significantly reduced the viability of N2a cells (*p* < 0.001), indicating that LPS‑induced microglial activation caused toxicity by excessive production of pro‑inflammatory mediators. As expected, increased cell viability was observed after treatment with CGPs‑1‑preconditioned medium (2.5‑CM, 5‑CM, 10‑CM), implying that CGPs‑1 could reverse the toxicity induced by microglial‑derived inflammatory mediators and exhibit cytoprotective potential against inflammation‑triggered neuronal damage in vitro.

### 2.11 Inhibitory Activity on Brain Inflammation in Zebrafish

MPTP (1‑methyl‑4‑phenyl‑1,2,3,6‑tetrahydropyridine) is a well‑known neurotoxin for constructing Parkinson's‑disease‑related animal models, which triggers cerebral neuroinflammation, activates inflammatory cells, releases inflammatory mediators and damages dopaminergic neurons [36]. To assess the anti‑inflammatory potential of CGPs‑1, *Tg (mpeg1: EGFP)* transgenic zebrafish were employed and cerebral inflammation was induced by MPTP treatment. As shown in Figure 8, compared with the blank control group, the fluorescence intensity in the MPTP group was significantly elevated (*p* < 0.001), indicating successful establishment of cerebral inflammatory injury in zebrafish. Compared with the model group, the fluorescence intensity was significantly decreased in the dexamethasone (DXM) positive‑control group and all CGPs‑1 treated groups (all *p* < 0.01). The inflammation‑inhibitory rate of dexamethasone (DXM) reached 70.12%, while the inhibitory rates for CGPs‑1 at 0.1, 0.2, and 0.4 µg/mL were 30.2%, 59.37%, and 70.12%, respectively. Collectively, these results demonstrated that CGPs‑1 exerted obvious inhibitory effects on MPTP‑provoked cerebral inflammatory responses in zebrafish model.

**FIGURE 8:**
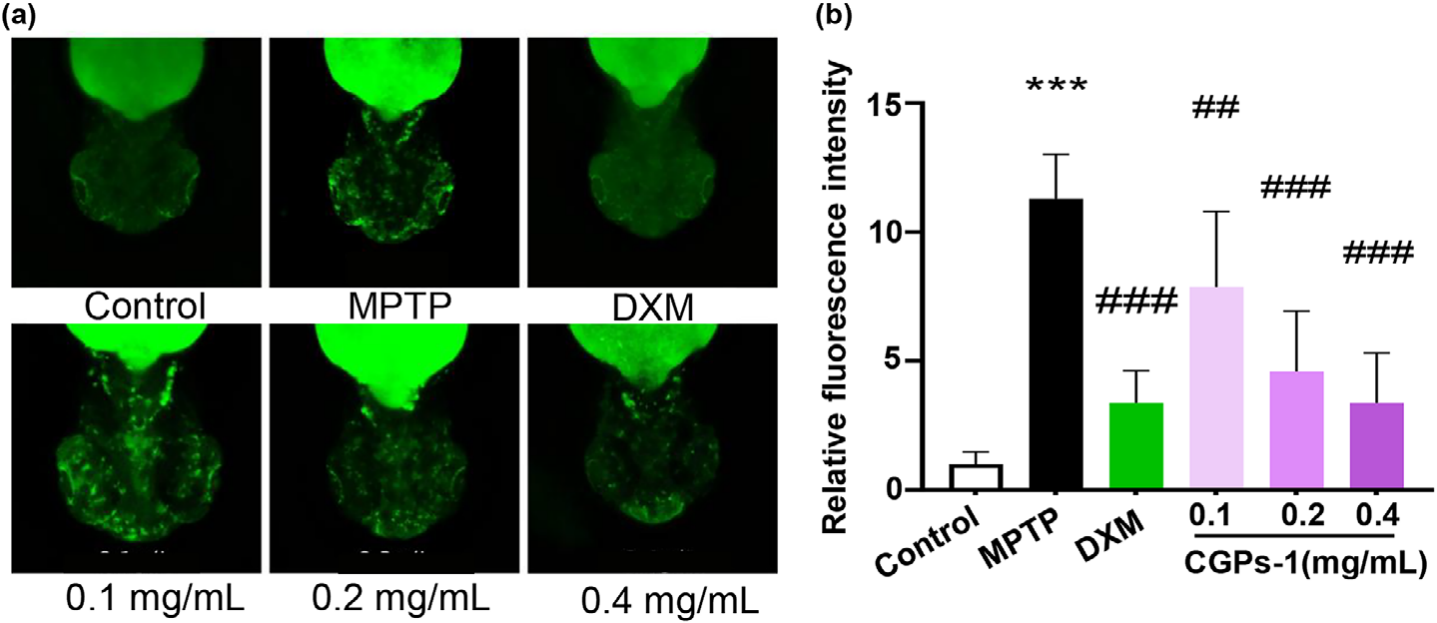
Inhibitory activity of CGPs‑1 on neuroinflammation in zebrafish. (a) Graph of zebrafish brain inflammation infiltration; (b) Quantitative analysis of fluorescence. *p < 0.05, **p < 0.01, ***p < 0.001 vs. control group; ^#^ *p* < 0.05, ^##^ *p* < 0.01, ^###^ *p* < 0.001 vs. MPTP group.

The consistent anti‑cerebral inflammatory phenotype across in‑vitro cellular and larval zebrafish models suggested that CGPs‑1 could mitigate inflammation‑mediated neuronal injury at both cellular and whole‑organism levels. Notably, two key factors account for the observable central protective effects in zebrafish larvae despite the macromolecular nature of CGPs‑1. First, the blood–brain barrier of early**‑**stage zebrafish larvae remains immature and highly permeable to macromolecules [37, 38], differing greatly from the compact barrier of adult mammals. Second, CGPs‑1 may alleviate cerebral inflammation indirectly by suppressing systemic circulating pro‑inflammatory mediators [39], consistent with the anti‑inflammatory activity observed in the preceding cell assays (Figure 7). These two mechanisms collectively explain the reduced microglial fluorescence signals in *mpeg1‑*transgenic zebrafish without conflicting with the inherent permeability limitation of high‑molecular‑weight polysaccharides.

## 3 Conclusion

In the present study, a homogeneous polysaccharide from sword bean pods (CGPs‑1) was obtained using ultrasound‑assisted DES method. The optimal extraction conditions were determined as: DES of ChCl‑OA (2:1), DES water content of 34%, liquid‑to‑solid ratio of 21 mL/g, temperature of 65°C, ultrasonic time of 29 min, and ultrasonic power of 280 W. Under these conditions, the actual polysaccharide yield reached 33.24%, close to the predicted value of 33.96%, and much higher than 28.67% obtained by ultrasound‑assisted water extraction under identical conditions. The purified CGPs‑1 is a pyran‑type polysaccharide composed of rhamnose (Rha), glucuronic acid (GlcA), and galacturonic acid (GalA) with a molar ratio of 1.08:1:1.78. Bioassays revealed its potential for antioxidant effects with DPPH• radical‑scavenging IC_50_ of 31.18 mg/mL and ABTS^+^• radical‑scavenging IC_50_ of 2.264 mg/mL, as well as hypoglycemic‑related potential via inhibiting α‑glucosidase (IC_50_: 0.157 mg/mL) and α‑amylase (IC_50_: 0.083 mg/mL). Furthermore, our research findings demonstrate that CGPs‑1 could not only inhibit LPS‑triggered inflammatory response and the associated LPS‑mediated neurotoxicity in BV2 cells but also suppress the MPTP‑induced inflammatory response in zebrafish larvae. These findings indicate that CGPs‑1 possesses antioxidant, hypoglycemic, anti‑neuroinflammatory and neuroprotective potentials, which may contribute to the intervention of hyperglycemia‑, oxidative stress‑ and neuroinflammation‑related neurodegenerative processes. This work provides preliminary experimental support for further research and the development of functional‑food‑related products derived from sword bean pods. Nevertheless, our understanding of CGPs‑1 remains incomplete: mammalian in‑vivo evidence is absent, its structural characterization remains preliminary, and the precise mechanisms underlying its anti‑neuroinflammatory effects remain unclear. Accordingly, future work should conduct mammalian in‑vivo validation of CGPs‑1 bioactivities, accomplish fine‑structural analysis to interpret its structure‑activity relationship, and decipher the mechanisms responsible for its anti‑neuroinflammatory effects.

## 4 Experimental Section

### 4.1 Materials and Reagents

The dried cuttings of sword bean (*Canavalia gladiata* (Jacq.) DC.) pods were commercially purchased from an online supplier (Liuxiang Xiancang), with product‑indicated origin in Hunan, China. Botanical identity was verified in‑house through morphological observation of the dried material upon receipt. The dried pod fragments were further crushed into fine powder before use, and residual unused raw materials (both cut fragments and powder) have been preserved in our laboratory sample repository. The following chemicals were used: phenol reagent and concentrated sulfuric acid, trifluoroacetic acid from Guangzhou Chemical Reagent (Guangzhou, China); DEAE‑cellulose from Huiyuan Biologicals (Quanzhou, China); Sephadex G‑75 from Xi'an Lanshao Science and Technology New Material Co., Ltd. (Shanxi, China); acarbose hydrate, PNPG (*p‐*nitrophenyl‐α‐d‐glucoside) and N‐Phenylthiourea (PTU) were from Aladdin Biochemical Technology Co., Ltd. (Shanghai, China); α‐glucosidase and anhydrous glucose from Genye Biotech (Guangzhou, China); α‐amylase, DPPH (1,1‐diphenyl‐2‐picrylhydrazyl), ABTS (2,2'‐azino‐bis‐(3‐ethylbenzothiazoline‐6‐sulfonic acid)) and PMP (1‐Phenyl‐3‐methyl‐5‐pyrazolone) from Maclean Biotechnology (Tianjin, China); L‐arabinose, D‐mannose, d‐(+)‐xylose, d‐(+)‐galacturonic acid, d‐galactose, d‐glucuronic acid, d‐(+)‐glucose anhydrous, rhamnose from Genuine Leaf Bio (Guangzhou, China); MPTP (1‐methyl‐4‐phenyl‐1,2,3,6‐tetrahydropyridine) was purchased from Sigma‐Aldrich Corporation (USA). LPS was purchased from Biosharp (Hefei, China), Dexamethasone (DXM) was from Solarbio Science & Technology Co., Ltd. (Beijing, China). Cell Counting Kit‐8 (CCK‐8) was purchased from APExBIO Technology LLC (USA). BV2 cells (mouse microglial cell line, RRID: CVCL_0182) was purchased from Procell Life Biotechnology (WuHan, China), Neuro‑2a (commonly abbreviated as N2a, mouse neuroblastoma‑derived neuron‑like cell line, RRID:CVCL_C5RG) was purchased from Jinio Biotechnology (Guangzhou, China). Enzyme‐linked immunosorbent assay (ELISA) kits for IL‐1β and IL‐6 were purchased from Elabscience Biotechnology Co., Ltd (Wuhan, China). Other reagents were from Tianjin Zhiyuan Chemical Reagent Co., Ltd. (Tianjin, China).

### 4.2 Preparation of Glucose Standard Curve

The glucose standard curve was established by the phenol‐sulfuric acid method with UV spectrophotometry according to a previous report [40], with minor modifications. Briefly, 4 mg of anhydrous glucose was used to prepare a 0.4 mg/mL standard solution. Aliquots of 10, 20, 30, 40, and 50 µL were pipetted into separate tubes, diluted to 100 µL with distilled water, followed by addition of 0.1 mL of 5% phenol and 0.5 mL of concentrated sulfuric acid. The mixture was vortex‐mixed and incubated in a boiling‐water bath for 15 min. An aliquot (200 µL) of the reaction mixture was transferred to a 96‐well plate, and the absorbance was measured at 492 nm. All measurements were performed in triplicate, and the standard curve was finally drawn.

### 4.3 Extraction Procedure and Polysaccharide Yield Calculation

The extraction was performed according to a previous report [41] with minor modifications. Briefly, 0.5 g of sword bean pod powder was accurately weighed and mixed with the selected DES, followed by ultrasound‐assisted extraction using an ultrasonic bath (KQ‐400KDB, Kunshan Ultrasonic Instruments Co., Ltd., Kunshan, China). The extraction was carried out at a constant temperature maintained by the built‐in temperature control function of the instrument, to avoid any temperature rise induced by ultrasonic cavitation. Then, the mixture was centrifuged at 4000 r/min for 5 min to separate the supernatant from solid residues. The polysaccharide content in the supernatant was determined, and the supernatant was subsequently concentrated.

Then 95% ethanol was added under stirring until the final ethanol concentration reached 80%. After standing for 48 h, the precipitate was collected by centrifugation and redissolved in 450 mL of distilled water. Protein impurities were removed via the Sevag method (polysaccharide solution: dichloromethane: *n*‐butanol = 25:4: 1, v/v), followed by another centrifugation step. The supernatant was further concentrated and dialyzed in flowing water for 48 h. After dialysis, the solution was centrifuged, and the resulting supernatant was concentrated and evaporated to dryness to obtain the crude sword bean polysaccharide. The polysaccharide concentration of samples was quantified using the phenol‐sulfuric acid method described above.

The polysaccharide yield was calculated according to Equation (2):

(2) Polysaccharideyield(%)=C×V/M×100%

where *C* is the polysaccharide concentration of the solution, *V* is the volume of the polysaccharide solution, and *M* is the mass of raw powder used for extraction.

### 4.4 Screening of Deep Eutectic Solvents

According to a previous report [42] with minor modifications, DES components were weighed according to the corresponding molar ratios and melted in an 80°C water bath under stirring until homogeneous transparent liquids were formed. Polysaccharides were extracted using seven different DES systems (Figure 1) under fixed conditions: water content of 30%, liquid‐to‐solid ratio of 20 mL/g, ultrasonic power of 280 W, ultrasonic time of 30 min, and temperature of 65. The polysaccharide yield for each DES was determined. All extraction experiments were conducted in triplicate. The DES system producing the highest polysaccharide yield was selected for subsequent experiments.

### 4.5 Single‐Factor Experiments

The effects of six factors—DES molar ratio, water content, temperature, liquid‐to‐solid ratio, ultrasonic power, and ultrasonic time—on the polysaccharide yield of sword bean polysaccharides was assessed using a single‐factor experimental design. Briefly, the baseline conditions (liquid‑to‐solid ratio 20 Ml/g, DES molar ratio 2:1, ultrasonic power 280 W, ultrasonic time 30 min, temperature 65 °C) were employed to sequentially investigate each variable: DES water content (0%, 10%, 20%, 30%, 40%, 50%), DES molar ratios (1:1, 1.5:1, 2:1, 2.5:1, 3:1), extraction temperatures (25°C, 35°C, 45°C, 55°C, 65°C, 75°C), ultrasonic power (160, 200, 240, 280, 320, 360 W), ultrasonic time (10, 30, 50, 70, 90, 110, 130 min), and liquid‐to‐solid ratios (5, 10, 15, 20, 25, 30 mL/g). After each factor was tested, the optimal level yielding the highest polysaccharide yield was adopted as the baseline for the next‑round test. All experiments were performed in triplicate, and the obtained optimal levels were selected for the subsequent Box‐Behnken design.

### 4.6 Response Surface Experiment

Based on the results of the single‐factor experiment, four significant factors with three levels each were selected (Table 3), and polysaccharide yield was set as the response value. The Box‐Behnken design was implemented using Design‑Expert 13.0 software, generating a total of 29 experimental runs. During response‑surface experiments, the DES molar ratio was fixed at 2:1 and extraction temperature was maintained at 65°C for determining polysaccharide yield. Finally, the predicted optimal extraction conditions were validated through confirmatory experiments.

**TABLE 3:** Response surface factors and levels.

Factor	Level		
	−1	0	1
A:Water content (%)	20	30	40
B:Liquid‐to‐solid ratio (mL/g)	15	20	25
C:Ultrasonic power (W)	240	280	320
D:Ultrasound time (min)	10	30	50

### 4.7 DEAE‐Sepharose Chromatography

The procedure was slightly modified from a previous report [40]. Crude sword bean polysaccharides were dissolved in distilled water to prepare a 25 mg/mL solution, followed by centrifugation at 4000 r/min for 5 min. 10 mL of the supernatant was loaded onto the pre‐equilibrated DEAE‐Sepharose Fast Flow column. The column was first eluted with 500 mL distilled water and subsequently eluted with 800 mL of a linear sodium chloride gradient (0–0.8 M) at a flow rate of 2 mL/min. Eluate fractions were collected at 10 mL per tube, and polysaccharide content was monitored by measuring A492 nm using the phenol‐concentrated sulfuric acid method. The elution curve was plotted. Peak‐containing fractions were pooled, dialysed, concentrated, and lyophilized to obtain purified sword‐bean polysaccharides.

### 4.8 Sephadex G‐75 Chromatography

4 mL of a 10 mg/mL polysaccharide solution (from the previous DEAE‑Sepharose step) was loaded onto the pre‑equilibrated Sephadex G‐75 column, which was eluted with 500 mL distilled water at a flow rate of 0.8 mL/min. Fractions were collected at 8 mL per tube, and polysaccharide content was monitored by measuring absorbance at 492 nm using the phenol‐sulfuric acid method. The elution curve was plotted. Peak‑containing fractions were pooled, dialysed, concentrated and lyophilized to yield the purified sword‑bean polysaccharide (CGPs‑1).

### 4.9 Molecular‐Weight Determination

The molecular weight of CGPs‑1 was determined by high‑performance gel‑permeation chromatography (HPGPC). Briefly, CGPs‑1 was dissolved in ultrapure water and filtered through a 0.22 µm aqueous‑phase membrane. The sample solution was injected onto a TSK‑Gel G4000 PWXL column (7.8 mm × 300 mm). Ultrapure water served as the mobile phase with a flow rate of 0.6 mL/min, and the column temperature was maintained at 35 °C. A refractive‑index detector (RID) was used for signal detection. A series of dextran standards with known molecular weights were applied to establish the standard calibration curve. The weight‑average molecular weight (Mw) of CGPs‑1 was calculated according to the calibration curve.

### 4.10 Monosaccharide Composition Determination

The monosaccharide composition of CGPs‑1 was analyzed by HPLC after trifluoroacetic acid (TFA) hydrolysis and 1‑phenyl‑3‑methyl‑5‑pyrazolone (PMP) derivatization, according to a previous report [43] with minor modifications.

Briefly, CGPs‑1 was hydrolyzed with TFA at 120°C for 4 h. The resulting hydrolysate was concentrated in a 50 mL flask at 65°C under vacuum. Methanol was added and evaporated three times to remove TFA. The dried solid was dissolved in 1 mL of deionized water. Then, 100 µL of hydrolysis solution, 0.5 M PMP‐methanol solution, and 0.3 M NaOH solution were mixed in a 5 mL centrifuge tube, and incubated in a water bath at 70°C for 30 min. After cooling, the mixture was neutralized with 105 µL of 0.3 M hydrochloric acid, diluted with 400 µL of deionized water, and extracted with 2 mL of chloroform. The chloroform layer was discarded, and the extraction step was repeated twice to remove excess PMP. The aqueous layer was then filtered through a 0.45 µm microporous membrane prior to injection.

A 10 µL aliquot of sample or standard solution was injected into an Agilent 1260 HPLC system equipped with a Kromasil 100‐5‐C18 column (5 µm, 4.6 × 250 mm). The column temperature was maintained at 25°C, and the detection wavelength was set at 250 nm. The mobile phase consisted of 85% 0.05 M phosphate buffer (pH 6.7) and 15% acetonitrile at a flow rate of 1 mL/min. Glucose (Glc), mannose (Man), arabinose (Ara), xylose (Xyl), galacturonic acid (GalA), glucuronic acid (GlcA), rhamnose (Rha), and galactose (Gal) were used as monosaccharide standards for qualitative and quantitative analysis.

### 4.11 Fourier‐Transform Infrared (FTIR) Spectroscopy‐

Briefly, 2 mg of CGPs‐1 was mixed with 200 mg of KBr, and ground thoroughly under dry conditions. The mixture was pressed into KBr tablets. FTIR spectra were recorded on a Fourier transform infrared spectrophotometer (Perkin Elmer, USA) over the wavenumber range of 4000–400 cm^−^^1^, using a pure KBr tablet as the background.

### 4.12 DPPH Radical‐Scavenging Assay

The assay was carried out according to a previous report [44] with minor modifications. Briefly, 100 µL sample solution at various concentrations (6400, 3200, 1600, 800, 400, 200, 100, 50, 25, 12.5 µg/mL) was mixed with 100 µL of 150 µM DPPH radical solution (prepared in absolute ethanol) in a 96‐well plate. The mixture was incubated at 37°C for 30 min in the dark, and the absorbance was measured at 517 nm. Vitamin C (Vit C) was used as positive control, and distilled water was used as negative control. For sample blank, 100 µL of absolute ethanol was added instead of DPPH working solution for each sample concentration. All determinations were performed in triplicate. The radical‑scavenging rate was calculated according to the following formula:

(3) $$\mathrm{Scavenging}\;\mathrm{rate}\;\left(\%\right)=[1-\left(A_{1}-A_{2}\right)/A_{0}]\;\;\times \;\;100\%$$

where *A*_1_ is the absorbance of the sample or Vit C reaction mixture; *A*_2_ is the absorbance of sample blank; *A*_0_ is the absorbance of negative‑control blank.

### 4.13 ABTS Radical‐Scavenging Assay

The assay was carried out according to a previous report [45] with minor modifications. ABTS was dissolved in distilled water at 7.4 mM and then oxidized to ABTS•^+^ radical cation by mixing with an equal volume of 2.6 mM potassium persulfate solution. The mixture was kept overnight at room temperature in the dark. The obtained ABTS•^+^ stock solution was diluted with absolute ethanol until its absorbance reached A_734nm_ = 0.70 ± 0.02. Afterwards, 40 µL Sample solution of different concentrations (6400, 3200, 1600, 800, 400, 200, 100, 50, 25, 12.5 µg/mL) was mixed with 160 µL diluted ABTS•^+^ working solution. After incubation for 30 min at room temperature in the dark, absorbance was recorded at 734 nm. Vit C was used as the positive control and distilled water as the negative control. For sample blank, 160 µL of absolute ethanol was added instead of ABTS•^+^ working solution for each sample concentration. All determinations were performed in triplicate. The scavenging rate (%) was calculated using the same formula described for the DPPH assay (Equation 3).

### 4.14 α‐Glucosidase Inhibition Assay

The assay was carried out according to a previous report [46] with minor modifications. Briefly, 40 µL of sample solution at various concentrations (6400, 3200, 1600, 800, 400, 200, 100, 50, 25, 12.5 µg/mL) were pre‐incubated with 40 µL of 1 U/mL α‐glucosidase in a 96‐well plate at 37°C for 10 min. Subsequently, 20 µL of 3 mM PNPG substrate solution was added, and the mixture was incubated at 37°C for 30 min. Finally, 100 µL of 1 M Na_2_CO_3_ solution was added to terminate the reaction. The absorbance was measured at 405 nm. Acarbose was used as the positive control. All determinations were performed in triplicate. The inhibition rate was calculated according to the following formula:

(4) $$\mathrm{Inhibition}\;\mathrm{rate}\;\left(\%\right)=\left(1-A_{1}/A_{2}\right)\times 100\%$$

where *A*_1_ is the absorbance of the sample or acarbose reaction mixture, *A*_2_ is the absorbance of enzyme control without sample.

### 4.15 α‐Amylase Inhibition Assay

The assay was carried out according to a previous report [46] with minor modifications. Briefly, 40 µL of sample solutions of different concentrations (6400, 3200, 1600, 800, 400, 200, 100, 50, 25 12.5 µg/mL, 40 µL) were pre‐incubated with 40 µL of 0.5 mg/mL α‐amylase in a 96‐well plate at 37°C for 10 min. Then, 20 µL of 1% starch solution was added and incubated at 37°C for 10 min. The reaction was terminated by adding 100 µL of DNS reagent, followed by incubation at 100°C for 10 min. After cooling, the mixture was diluted with 1 mL of distilled water, and absorbance was determined at 540 nm. Acarbose was used as the positive control. All determinations were performed in triplicate. The inhibition rate (%) was calculated using the same formula described for the α‐glucosidase assay (Equation 4).

### 4.16 In Vitro Anti‐Inflammatory Assay

BV‑2 cells in logarithmic growth phase were seeded into 96‐well culture plates at a density of 3 × 10^4^ cells/well in 100 µL medium and incubated overnight. On the next day, cells were pre‐treated with CGPs‑1 at concentration of 2, 5, and 10 µg/mL for 2 h; an untreated group was set as control. Afterwards, A final concentration of 1 µg/mL LPS was added, and cells were further incubated for 24 h. An equal volume of culture medium without cells were set as the blank group, and untreated cells served as the control group. Subsequently, the supernatant was replaced with 100 µL medium containing 10% CCK‐8 reagent, followed by incubation at 37°C for 45 min. Absorbance at 450 nm was measured using a Multiskan FC microplate reader (Thermo Fisher Scientific), and cell viability (%) was calculated according to the following formula:

(5) $$\mathrm{Cell}\;\mathrm{viability}\;\left(\%\right)=[\left(A_{1}-A_{0}\right)/\left(A_{2}-A_{0}\right)]\;\times 100\%$$

where *A*_1_ is the absorbance of treated groups; *A*_2_ is the absorbance of the control group; *A*_0_ is the absorbance of the blank group.

The level of nitric oxide (NO) in cell supernatants was determined by the Griess method following the NO assay kit instructions. The levels of pro‐inflammatory cytokines (IL‐1β, IL‐6, and TNF‐α) were quantified using corresponding enzyme‐linked immunosorbent assay (ELISA) kit instructions according to the manufacturer's protocols.

N2a cells in logarithmic growth phase were seeded into 96‐well culture plates at a density of 5 × 10^4^ cells/well in 100 µL medium and incubated overnight. The culture medium was then replaced with the conditioned medium (CM) collected from LPS‑stimulated BV2 cells with or without CGPs‑1 pre‑treatment, and N2a cells were for 24 h. Thereafter, 100 µL of medium containing 10% CCK‐8 reagent was added. After 45 min incubation at 37°C, absorbance was recorded at 450 nm. Cell viability (%) was calculated using the same formula as described above (Equation 5).

### 4.17 In Vivo Anti‐Neuroinflammatory Assay

All experimental protocols and procedures were conducted in strict accordance with the Laboratory Animal Management Regulations of Guangdong Pharmaceutical University, under animal ethical approval number [gdpulac2025410]. Transgenic Tg *(mpeg1:EGFP)* Zebrafish embryos (Guangzhou Shanhai Biotechnology Co., Ltd.) were used at 24 h post‐fertilization (hpf), prior to the stage of independent feeding. Experiments were performed in 24‐well plates, with 10 embryos per well and three replicate wells per group. Holtfreter's buffer (HB) served as the basal medium, containing 60 mM NaCl, 20 mM NaHCO_3_, 1.35 mM KCl, and 1.25 mM CaCl_2_. To prevent skin pigmentation and facilitate fluorescence observation, embryos were treated with 0.003% 1‐phenyl‐2‐thiourea (PTU, Sigma, w/v%) starting from 24 hpf. Each well was filled with 2 mL of working solution in HB. Four experimental groups were set: control (Ctrl) group (0.003% PTU), model group (50 µM MPTP + 0.003% PTU), positive‑drug group (50 µM MPTP + 0.003% PTU + 40 µM DXM), and polysaccharide‑treatment groups (50 µM MPTP + 0.003% PTU + CGPs‐1 at gradient concentrations of 0.1, 0.1, 0.4 g/L). All working solutions used were also freshly prepared with HB. Embryos were incubated at 28°C ± 1°C for 48 h under complete darkness. When embryos reached 72 hpf, they were mounted in 3 % methylcellulose with heads oriented upward. Fluorescence imaging of zebrafish brain regions was captured by an upright fluorescence microscope using a 10× objective lens. The fluorescence intensity within brain regions was quantified by ImageJ software, and GraphPad Prism 10 was used for plotting graphs and statistical analysis.

## Statistical Analysis

Data collected from three replicate experiments are expressed as the mean ± standard deviation. Statistical differences between two groups were determined using Student's *t‐*test, while comparisons among three or more groups were performed by one‐way analysis of variance (ANOVA) followed by Tukey's post‑hoc test. A *p*‐value of less than 0.05 was considered as statistically significant.

## Author Contributions

**Jieyu Xing** and **Linjing Zhang**: investigation, analysis, the drafting and revision of the initial manuscript. **Zhaoxuan Liang**: investigation. **Zheng Liu**: revision of the manuscript. **Haiyun Chen** and **Xian Fu**: core ideas, guidance for the research, supervision, and revision of the manuscript.

## Ethics Statement

All experimental protocols and procedures involving zebrafish embryos were conducted in strict accordance with the Laboratory Animal Management Regulations of Guangdong Pharmaceutical University. Ethical approval was obtained from the Animal Ethics Committee of Guangdong Pharmaceutical University (approval number: gdpulac2025410). All experiments were performed in compliance with the ARRIVE guidelines and the 3Rs principle (Replacement, Reduction, Refinement) for animal research.

## Conflicts of Interest

The authors declare no conflicts of interest.

## Data Availability

The data that support the findings of this study can be obtained from the corresponding author upon reasonable request.

## References

[1] J. X. Huai , E. E. Chang , Y.‐R. Zhu , et al., “Diabetic Encephalopathy: Metabolic Reprogramming as a Potential Driver of Accelerated Brain Aging and Cognitive Decline,” Frontiers in Cell and Developmental Biology 13 (2025): 1701406, 10.3389/fcell.2025.1701406. PMC12665671 41332987

[2] A. Raza , S. Saleem , S. Imran , et al., “From Metabolic Dysregulation to Neurodegenerative Pathology: The Role of Hyperglycemia, Oxidative Stress, and Blood–Brain Barrier Breakdown in T2D‐Driven Alzheimer's Disease,” Metabolic Brain Disease 40 (2025): 276, 10.1007/s11011-025-01700-z. PMC12474712 41003796

[3] D. H. Bach and T. L. Nguyen , “Immunometabolism Reframes Alzheimer's Disease: From Systemic Dysmetabolism to Glial Rewiring,” Cellular and Molecular Neurobiology 46 (2026): 56, 10.1007/s10571-026-01691-0. PMC12957698 41688855

[4] T. Ponce‐Lopez , “Peripheral Inflammation and Insulin Resistance: Their Impact on Blood–Brain Barrier Integrity and Glia Activation in Alzheimer's Disease,” International Journal of Molecular Sciences 26 (2025): 4209, 10.3390/ijms26094209. PMC12072112 40362446

[5] Q. H. Gao , X. Fu , R. Zhang , Z. Wang , and M. Guo , “Neuroprotective Effects of Plant Polysaccharides: A Review of the Mechanisms,” International Journal of Biological Macromolecules 106 (2018): 749–754, 10.1016/j.ijbiomac.2017.08.075. 28818727

[6] R. Guo , J. Pang , J. Zhao , et al., “Unveiling the Neuroprotective Potential of Dietary Polysaccharides: A Systematic Review,” Frontiers in Nutrition 10 (2023): 1299117, 10.3389/fnut.2023.1299117. PMC10702503 38075226

[7] R. Chen , J. Xu , W. Wu , et al., “Structure–Immunomodulatory Activity Relationships of Dietary Polysaccharides,” Current Research in Food Science 5 (2022): 1330–1341, 10.1016/j.crfs.2022.08.016. PMC9445227 36082139

[8] Y. Li , Y. Liu , J. Xu , Y. Zhang , L. Zhao , and Y. Miao , “Artificial Neural Network Techniques to Predict the Moisture Ratio Content During Hot Air Drying and Vacuum Drying of *Radix isatidis* Extract,” Traditional Medicine Research 7 (2022): 5, 10.53388/TMR20210916244.

[9] W. Y. Huang , S. H. Lee , S. J. Oh , et al., “ *Canavalia gladiata* Pod Extract Mitigates Ovalbumin‐Induced Asthma Onset in Male BALB/c Mice via Suppression of MAPK,” Molecules 27 (2022): 6317, 10.3390/molecules27196317. PMC9573573 36234854

[10] H. J. Lee , J. U. Park , R. H. Guo , B. Y. Kang , I. K. Park , and Y. R. Kim , “Anti‐Inflammatory Effects of *Canavalia gladiata* in Macrophage Cells and DSS‐Induced Colitis Mouse Model,” American Journal of Chinese Medicine 47 (2019): 1571–1588, 10.1142/S0192415X19500800. 31645121

[11] K. A. Hwang , Y. J. Hwang , H. J. Hwang , S. H. Lee , and Y. J. Kim , “Sword Bean (*Canavalia gladiata*) Pod Exerts Anti‐Allergic and Anti‐Inflammatory Effects Through Modulation of Th1/Th2 Cell Differentiation,” Nutrients 14 (2022): 2853, 10.3390/nu14142853. PMC9322724 35889810

[12] H. J. Hwang , Y. J. Hwang , Y. J. Kim , M. Kim , and K. A. Hwang , “Immature Sword Bean Pods (*Canavalia gladiata*) Inhibit Adipogenesis in C3H10T1/2 Cells and Mice With High‐Fat Diet–Induced Obesity,” Journal of the Chinese Medical Association 85 (2022): 67–76, 10.1097/JCMA.0000000000000655. PMC12755440 34966163

[13] J. W. Hong , H. Y. Park , H. A. Kim , Y. S. Hwang , E. J. Lee , and J. W. Kim , “Inhibition Effect of Adipogenesis and Lipogenesis via Activation of AMPK in Preadipocytes Treated With *Canavalia gladiata* Extract,” International Journal of Molecular Sciences 24 (2023): 2108, 10.3390/ijms24032108. PMC9916869 36768430

[14] Y. J. Hwang , H. J. Hwang , H. Go , N. Park , and K. A. Hwang , “Sword Bean (*Canavalia gladiata*) Pods Induce Differentiation in MC3T3‐E1 Osteoblast Cells by Activating the BMP2/SMAD/RUNX2 Pathway,” Nutrients 15 (2023): 4372, 10.3390/nu15204372. PMC10610144 37892447

[15] R. Y. Gan , K. W. Kong , H. B. Li , et al., “Separation, Identification, and Bioactivities of the Main Gallotannins of Red Sword Bean (*Canavalia gladiata*) Coats,” Frontiers in Chemistry 6 (2018): 39, 10.3389/fchem.2018.00039. PMC5835520 29541634

[16] S. Une , K. Nonaka , and J. Akiyama , “Lectin Isolated From Japanese Red Sword Beans (*Canavalia gladiata* ) as a Potential Cancer Chemopreventive Agent,” Journal of Food Science 83 (2018): 837–843, 10.1111/1750-3841.14057. 29437230

[17] Y. Zhou , X.‐Y. Xu , R. Y. Gan , et al., “Optimization of Ultrasound‐Assisted Extraction of Antioxidant Polyphenols From the Seed Coats of Red Sword Bean (*Canavalia gladiata* (Jacq.) DC.),” Antioxidants 8 (2019): 200, 10.3390/antiox8070200. PMC6680378 31261608

[18] H. J. An , E. H. Kim , H. J. Lee , J. Y. Cho , and J. H. Moon , “New Caryophyllene‐Type Sesquiterpene and Flavonol Tetraglycoside With Sixteen Known Compounds From Sword Bean (*Canavalia gladiata*),” Food Science and Biotechnology 29 (2020): 1343–1353, 10.1007/s10068-020-00794-8. PMC7492346 32999741

[19] L. B. Vinh , S. H. Shin , Y. K. Han , et al., “Identification of Interleukin (IL)‐33 Inhibitory Constituents From *Canavalia gladiata* Pods,” Antioxidants 13 (2024): 767, 10.3390/antiox13070767. PMC11273637 39061836

[20] L. Zhang and M. Wang , “Optimization of Deep Eutectic Solvent‐Based Ultrasound‐Assisted Extraction of Polysaccharides From *Dioscorea opposita* Thunb,” International Journal of Biological Macromolecules 95 (2017): 675–681, 10.1016/j.ijbiomac.2016.11.096. 27908714

[21] Y. Wen , X. Yan , and H. Chen , “Optimization of Ultrasonic‐Assisted Deep Eutectic Solvent Extraction, Characterization, and Bioactivities of Polysaccharides From *Eucommia ulmoides* ,” Preparative Biochemistry & Biotechnology 55 (2025): 577–589, 10.1080/10826068.2024.2441914. 39892421

[22] W. Yang , S. Jiang , X. Wu , et al., “Ultrasound‐Assisted Deep Eutectic Solvent Extraction of Ginseng Folium Polysaccharides: Extraction Mechanism, Process Optimization, Structural Characterization, and In Vitro Biological Activity,” Ultrasonics Sonochemistry 127 (2026): 107797, 10.1016/j.ultsonch.2026.107797. PMC12966679 41762774

[23] L. Ke , X. Duan , J. Cui , et al., “Research Progress on the Extraction Technology and Activity Study of Epimedium Polysaccharides,” Carbohydrate Polymers 306 (2023): 120602, 10.1016/j.carbpol.2023.120602. 36746589

[24] Y. Gao , J. Tan , Y. Sang , J. Tang , X. Cai , and H. Xue , “Preparation, Structure, and Biological Activities of the Polysaccharides From Fruits and Vegetables: A Review,” Food Bioscience 54 (2023): 102909, 10.1016/j.fbio.2023.102909.

[25] S. Della Posta , V. Gallo , A. Gentili , and C. Fanali , “Strategies for the Recovery of Bioactive Molecules from Deep Eutectic Solvents Extracts,” TrAC Trends in Analytical Chemistry 157 (2022): 116798, 10.1016/j.trac.2022.116798.

[26] J. Osamede Airouyuwa , N. Sivapragasam , A. Ali Redha , and S. Maqsood , “Sustainable Green Extraction of Anthocyanins and Carotenoids Using Deep Eutectic Solvents (DES): A Review of Recent Developments,” Food Chemistry 448 (2024): 139061, 10.1016/j.foodchem.2024.139061. 38537550

[27] C. Wang , Q. Li , D. Qiu , Y. Guo , X. Ding , and K. Jiang , “An Efficient and Environmentally‐Friendly Extraction, Characterization and Activity Prediction of Polysaccharides From Rhizoma Et *Radix notopterygii* ,” International Journal of Biological Macromolecules 265 (2024): 130907, 10.1016/j.ijbiomac.2024.130907. 38492707

[28] M. Meenu , V. Bansal , S. Rana , et al., “Deep Eutectic Solvents (DESs) and Natural Deep Eutectic Solvents (NADESs): Designer Solvents for Green Extraction of Anthocyanin,” Sustainable Chemistry and Pharmacy 34 (2023): 101168, 10.1016/j.scp.2023.101168.

[29] X. Pan , L. Xu , J. Meng , et al., “Ultrasound‐Assisted Deep Eutectic Solvents Extraction of Polysaccharides From *Morchella importuna*: Optimization, Physicochemical Properties, and Bioactivities,” Frontiers in Nutrition 9 (2022): 912014, 10.3389/fnut.2022.912014. PMC9218490 35757264

[30] H. Zhang , J. Lang , P. Lan , H. Yang , J. Lu , and Z. Wang , “Study on the Dissolution Mechanism of Cellulose by ChCl‐Based Deep Eutectic Solvents,” Materials 13 (2020): 278, 10.3390/ma13020278. PMC7014281 31936299

[31] M. H. Md Yusoff and M. H. Shafie , “Pioneering Polysaccharide Extraction With Deep Eutectic Solvents: A Review on Impacts to Extraction Yield, Physicochemical Properties and Bioactivities,” International Journal of Biological Macromolecules 306 (2025): 141469. 10.1016/j.ijbiomac.2025.141469 40015410

[32] X. Sun , J. A. Zhang , R. Guo , X. Ma , L. Song , and Y. Wu , “Structural Features and Antioxidant Efficacy of a Novel Low‐Molecular‐Weight Pectic Polysaccharide From Fuzhuan Brick Tea,” Food Bioscience 79 (2026): 108805, 10.1016/j.fbio.2026.108805.

[33] H. Wang , Y. Li , Z. Ren , et al., “Optimization of the Microwave‐Assisted Enzymatic Extraction of *Rosa roxburghii* Tratt. Polysaccharides Using Response Surface Methodology and Its Antioxidant and α‐D‐Glucosidase Inhibitory Activity,” International Journal of Biological Macromolecules 112 (2018): 473–482, 10.1016/j.ijbiomac.2018.02.003. 29412175

[34] A. Moorthy , S. Govindan , A. Janardhanan , S. Srinivasan , H. Padinjarathil , and P. Ramani , “Response Surface Methodology Optimization Extraction of Polysaccharide From *Tricholoma lobayense* and Its Biological Activities,” Journal of Food Measurement and Characterization 18 (2024): 2133–2145, 10.1007/s11694-023-02295-8.

[35] H. Xiao , X. Cai , Y. Fan , and A. Luo , “Antioxidant Activity of Water‐soluble Polysaccharides From *Brasenia Schreberi* ,” Pharmacognosy Magazine 12 (2016): 193. 10.4103/0973-1296.186343 PMC4989794 27601849

[36] M. Xie , Y. Cheng , L. Yang , et al., “Tetramethylpyrazine‐Caffeic Acid Hybrid CT‐011 Exerts Dopaminergic Neuroprotection Through Inhibiting Microglia‐Mediated Neuroinflammation,” Molecular Neurobiology 63 (2025): 44, 10.1007/s12035-025-05310-9. 41239042

[37] A. Fleming , H. Diekmann , and P. Goldsmith , “Functional Characterisation of the Maturation of the Blood–Brain Barrier in Larval Zebrafish,” Public Library of Science ONE 8 (2013): e77548, 10.1371/journal.pone.0077548. PMC3797749 24147021

[38] N. M. O'Brown , S. G. Megason , and C. Gu , in Suppression of Transcytosis Regulates Zebrafish Blood–Brain Barrier Function (Elife, 2019), e47326. 10.7554/eLife.47326 PMC6726461 31429822

[39] J. Gao , L. Li , Q. Liu , N. Zhang , and Y. Li , “Plant Polysaccharides in Alzheimer's Disease: From Phytochemistry to Microbiota‐Gut–Brain Axis Mechanisms—Resolving the Pharmacokinetic–Pharmacodynamic Paradox,” Molecules 31 (2026): 2622, 10.3390/molecules31152622. PMC13468679 42588471

[40] C. Huang , X. Cao , X. Chen , et al., “A Pectic Polysaccharide From *Ligusticum chuanxiong* Promotes Intestine Antioxidant Defense in Aged Mice,” Carbohydrate Polymers 174 (2017): 915–922, 10.1016/j.carbpol.2017.06.122. 28821148

[41] B. Bai , Q. Li , and X. Ma , “Optimization of Ultrasound‐Assisted Deep Eutectic Solvent Extraction of Polysaccharides From Jujube,” Food Research and Development 43 (2022): 122.

[42] J. Yao , J. Y. Zeng , Y. X. Tang , et al., “Effects of the Extraction Solvents on Dissolution Rate and Antioxidant Capacity of *Auricularia auricula* (Agaricomycetes) Polysaccharides In Vitro and In Vivo,” International Journal of Medicinal Mushrooms 25 (2023): 61–74, 10.1615/IntJMedMushrooms.2023048033. 37183919

[43] X. D. Wu , W. Jiang , J. J. Lu , Y. Yu , and B. Wu , “Analysis of the Monosaccharide Composition of Water‐Soluble Polysaccharides from *Sargassum fusiforme* by High Performance Liquid Chromatography/Electrospray Ionisation Mass Spectrometry,” Food Chemistry 145 (2014): 976–983, 10.1016/j.foodchem.2013.09.019. 24128572

[44] Z.‐Z. Jiao , S. Yue , H. X. Sun , et al., “Indoline Amide Glucosides From *Portulaca oleracea*: Isolation, Structure, and DPPH Radical Scavenging Activity,” Journal of Natural Products 78 (2015): 2588–2597, 10.1021/acs.jnatprod.5b00524. 26562741

[45] A. C. Ruberte , S. Ramos‐Inza , C. Aydillo , et al., “Novel N,N′‐Disubstituted Acylselenoureas as Potential Antioxidant and Cytotoxic Agents,” Antioxidants 9 (2020): 55, 10.3390/antiox9010055. PMC7023466 31936213

[46] R. Vadivelan , R. Gopala Krishnan , and R. Kannan , “Antidiabetic Potential of Asparagus Racemosus Willd Leaf Extracts Through Inhibition of α‐Amylase and α‐Glucosidase,” Journal of Traditional and Complementary Medicine 9 (2019): 1–4, 10.1016/j.jtcme.2017.10.004. PMC6335566 30671360

